# Factors Determining Patient-Prosthesis Mismatch after Aortic Valve Replacement – A Prospective Cohort Study

**DOI:** 10.1371/journal.pone.0081940

**Published:** 2013-12-03

**Authors:** Diana Bonderman, Alexandra Graf, Andreas A. Kammerlander, Alfred Kocher, Guenter Laufer, Irene M. Lang, Julia Mascherbauer

**Affiliations:** 1 Department of Cardiology, Medical University of Vienna, Vienna General Hospital, Vienna, Austria; 2 Department of Medical Statistics, Medical University of Vienna, Vienna General Hospital, Vienna, Austria; 3 Department of Cardiothoracic Surgery, Medical University of Vienna, Vienna General Hospital, Vienna, Austria; Universityhospital Düsseldorf, Germany

## Abstract

**Objective:**

“Patient-prosthesis mismatch” (PPM) after aortic valve replacement (AVR) has been reported to increase morbidity and mortality. Although algorithms have been developed to avoid PPM, factors favouring its occurrence have not been well defined.

**Design and Setting:**

This was a prospective cohort study performed at the Medical University of Vienna.

**Patients:**

361 consecutive patients who underwent aortic valve replacement for isolated severe aortic stenosis were enrolled.

**Main Outcome Measures:**

Patient- as well as prosthesis-related factors determining the occurrence of moderate and severe PPM (defined as effective orifice area indexed to body surface area ≤ 0.8 cm^2^/m^2^) were studied.

**Results:**

Postoperatively, 172 patients (48%) were diagnosed with PPM. The fact that predominantly female patients were affected (58% with PPM diagnosis in women versus 36% in men, p<0.001) was explained by the finding that they had smaller aortic root diameters (30.5±4.7 mm versus 35.3±4.2 mm, p<0.0001) and a higher proportion of bioprosthetic valves (82% versus 62%, p<0.0001), both independent predictors of PPM (aortic root diameter: OR 0.009 [95% CI, 0.004;0.013]; p = 0.0003, presence of bioprosthetic valve: OR 0.126 [95% CI, 0.078;0.175]; p<0.0001).

**Conclusions:**

The occurrence of PPM is determined by aortic root diameter and prosthesis type. Novel sutureless bioprostheses with optimized hemodynamic performance or transcatheter aortic valves may become a promising alternative to conventional bioprosthetic valves in the future.

## Introduction

The phenomenon of “patient-prosthesis mismatch” (PPM) after aortic valve replacement (AVR) has recently gained great interest. PPM is defined as a small orifice area of the aortic valve prosthesis in relation to the body surface area (BSA) [1], [2]. It has been shown to be associated with worse hemodynamic function, faster degeneration of bioprosthetic prostheses [3], and less regression of left ventricular hypertrophy. In addition, an impaired exercise capacity and increased occurrence of arrhythmias has been reported to be associated with PPM [4]. A recent meta-analysis of 34 observational studies published in the European Heart Journal showed a significant reduction in overall and cardiac-related long-term survival for patients with PPM after AVR [5].

Despite a growing body of evidence linking the presence of PPM with adverse outcome, conditions favoring its occurrence have not been well defined. The description of such conditions, however, is of great importance given that PPM is a potentially modifiable risk factor. In theory, PPM is thought to occur for two reasons. First, patients with aortic valve disease frequently exhibit annulus calcification and fibrosis as well as left ventricular hypertrophy, and these pathologic processes can reduce the size of the aortic annulus. Second, the prosthesis inserted within the aorta has its own structural support that may create a relative obstruction to flow [6]. Narrowing of the effective orifice area (EOA) of the valve in conjunct with an unchanged transvalvular flow, which, at rest, is largely determined by BSA results in elevated transprosthetic pressure gradients.

In the present work, we evaluated the impact of patient- related parameters, such as aortic annulus diameter, left ventricular hypertrophy, body size, age and gender, as well as the influence of prosthesis type on the occurrence of PPM. In a cohort of consecutive patients referred to aortic valve replacement for isolated severe aortic stenosis (AS), PPM patients were older, more likely to be female and had smaller aortic annuli. However, only carriers of bioprosthetic valves and patients with smaller aortic root diameters were at increased risk for PPM in a multivariable regression analysis.

## Methods

### Study design

This was an observational single center study performed at the outpatient clinic for valvular heart disease of the Medical University of Vienna. Between January 1999 and January 2005, consecutive patients diagnosed with isolated severe AS subsequently referred for conventional AVR who agreed to participate were enrolled. Patients were not randomly assigned to receive valves of a particular size or type. Prosthesis choice was exclusively upon the discretion of the treating surgeon in accordance with current recommendations [7], [8]. To study potential predictors of PPM, clinical, echocardiographic, and operative data were prospectively collected in a computerized database.

Reference EOA values for each size and model of prosthesis used in our study population have previously been published [9] and are summarized in Table 1. According to published data [9], [10], PPM was diagnosed if EOA indexed to the patientś BSA was ≤ 0.8 cm^2^/m^2^.

**Table 1:** Patient characteristics according to prosthesis type and effective orifice area.

Prosthesis type	Size [mm] [cm^2^]	EOA [cm^2^]	Patients [n]	Men [n]	Age [years]	BSA [m^2^]	
**Stented bioprosthetic valves**							
Medtronic Mosaic [12]	21	1.22	26	5	75.6±5.3		1.78±0.21
	23	1.38	18	10	71.4±7.5		1.84±0.13
	25	1.65	8	7	74.1±7.2		1.95±0.18
	27	1.8	3	2	70.7±5.0		2.07±0.21
C-E pericardial [13]	19	1.1	15	0	75.8±4.1		1.75±0.14
	21	1.3	65	9	75.8±6.5		1.80±0.18
	23	1.5	40	28	75.3±5.7		1.92±0.20
	25	1.8	14	13	71.3±6.6		1.93±0.13
	27	1.8	3	3	65.0±4.6		1.97±0.05
C-E Perimount Magna [14]	19	1.35	5	0	75.2±3.96		1.75±0.21
	21	1.75	18	4	74.5±6.10		1.80±0.19
	23	2.19	13	9	71.2±5.90		1.97±0.21
	25	2.35	4	3	68.3±4.35		2.04±0.25
Sorin Soprano [15]	20	1.59	2	0	85.5±4.95		1.39±0.06
	22	1.82	5	1	71.0±9.14		1.83±0.33
	24	2.27	2	1	70.0±8.48		2.06±0.13
**Stentless bioprosthetic valves**							
St Jude Toronto SPV [16]	21	1.2	2	0	72.5±4.95		1.70±0.16
	23	1.59	4	4	74.3±4.92		1.85±0.15
	25	1.62	3	1	68.0±4.00		1.85±0.31
	27	2.00	1	1	74		1.88
Edwards Prima [16]	23	1.5	8	3	63.8±9.30		2.00±0.28
	25	1.7	4	1	63.0±2.94		1.85±0.11
	27	2.0	1	1	64		2.06
**Mechanical valves**							
Carbomedics [17]	19	1.0	2	0	69.5±14.9		1.72±0.17
	21	1.54	6	3	51.0±7.4		1.82±0.21
	23	1.63	14	12	56.2±9.5		1.97±0.18
	25	1.98	10	9	57.7±4.4		2.05±0.24
	27	2.41	2	2	53.0±8.5		2.04±0.25
ON-X [16]	19	1.5	2	0	54.5±13.4		1.81±0.02
	21	1.7	4	3	55.8±5.1		2.03±0.17
	23	2.0	9	7	57.4±5.7		1.95±0.19
	25	2.4	6	6	54.3±10.7		2.02±0.17
	27	3.2	1	1	54		2.16
Edwards Mira [18]	19	1.17	1	0	57		1.75
	21	1.93	0	0			
	23	2.43	6	6	58.7±7.6		2.00±0.17
	25	2.56	2	2	48.5±0.7		2.03±0.02
Medtronic Advantage [19]	21	1.65	5	0	70.0±5.8		1.65±0.26
	23	2.17	3	2	51.3±15.3		1.87±0.19
	25	2.80	3	2	54.7±10.3		2.05±0.16
Medtronic Hall [16]	21	1.08	3	0	65.3±10.1		1.73±0.16
	23	1.36	4	2	59.8±8.3		2.00±0.21
	25	1.9	3	1	63.0±4.4		1.97±0.29
	27	1.9	2	2	57.0±0.0		2.26±0.38
Sorin Bicarbon [16]	23	1.98	3	1	59.0±3.6		2.05±0.25
St Jude Med. Standard [16]	19	1.01	1	0	75.00		1.77
	21	1.33	2	0	67.5±2.1		1.89 ±0.16
	23	1.6	1	0	87		1.69
Carbomedics Top Hat [16]	21	1.18	2	2	57.5±6.4		2.12±0.17

Size, nominal size of the prosthetic valve; EOA, reference effective orifice area; BSA, body surface area.

### Ethics statement

All data were collected prospectively. According to the study design (non-interventional, purely observational study) written informed consent was not demanded. Verbal agreement of the patients to participate was documented in the medical records. The ethics committee of the Medical University of Vienna approved the present study “Ergebnisse nach Herzklappenoperationen” (engl. “Results after Heart Valve Surgery”).

### Inclusion criteria

Patients were referred to AVR, if they suffered from isolated severe AS (peak velocity ≥4 m/s and mean pressure gradient ≥40 mmHg in the presence of normal left ventricular function, calculated valve area <1.0 cm^2^) and presented with symptoms (exertional dyspnea ≥ functional NYHA class II and/or exertional angina pectoris ≥ functional CCS class II and/or syncope) or with reduced left ventricular function (ejection fraction ≤50%) when asymptomatic. Patients with a need for additional coronary artery bypass grafting (CABG) at the time of aortic valve replacement were not excluded from the study. However, those who were primarily referred to surgery for CABG and additionally received an aortic prosthesis for non-severe AS were not enrolled. Patients with more than mild concomitant aortic regurgitation were also excluded.

### Patient evaluation

The following baseline data were collected: age, gender, weight and height. Further assessment at study entry included medical history, physical examination and transthoracic echocardiography with Doppler measurements. All left ventricular (LV) and aortic measures were quantified according to current recommendations [11]. Echocardiographic studies were performed by board certified physicians in the echo laboratory of the Division of Cardiology of the Medical University of Vienna, using high-end scanners, such as Siemens Acuson Sequoia and GE Vivid 5 and Vivid 7.

### Statistical analysis

Comparison of baselines variables was done by comparison of means (T-test) or by comparison of frequencies (Chi-square test). Kaplan-Meier estimates were used to calculate 2, 4, 6, 8, 10, and 12-year survival rates. Differences between survival curves were analyzed using log rank test from PROC LIFETEST. Differences in these and all other tests were considered significant at p ≤ 0.05.

To identify factors influencing prosthesis size (model 1) or EOA/BSA (model 2), separate simple regression models were calculated. All co-variables that showed a significant influence (p<0.05, 2-sided) on the main target variable (prosthesis size in model 1 or EOA/BSA in model 2) were included into a multiple regression model with backward selection.

The following influence variables were considered:

Metric: age, BSA (only model 1), valve area by the Doppler continuity equation, mean pressure gradient across the stenosis, aortic annulus diameter, LV wall thickness, LV diameter, aortic root diameter measured at the sinotubular junction

Dichotomous: gender, mechanical or biological valve replacement

As part of the main target variable (EOA/BSA), BSA was excluded from the analysis in model 2.

For sensitivity analysis, simple and multiple logistic regression models with backward selection were performed for the binary outcome variable PPM defined as EOA/BSA ≤ 0.8 cm^2^/m^2^, accounting for the same influence factors as shown above. No interactions were included in the model.

To investigate correlations between influence variables, Pearson correlation coefficients and univariable tests were performed.

Statistical analysis was performed using SAS 9.1 for Windows (SAS statistical software, SAS Institute, Cary, NC, USA).

## Results

### Baseline characteristics

361 patients fulfilled the inclusion criteria, of those 75% underwent isolated AVR and 25% received AVR with CABG. A majority of patients received bioprosthetic valves (n = 261), and 100 patients mechanical valve prostheses. Table 1 lists demographic characteristics of study patients according to prosthesis type and size. Patients with bioprosthetic valves were significantly older (73.7±6.9 years) compared with mechanical valve carriers (58.3±8.9 years, p<0.0001) and were more likely to be female (60% versus 35%, p<0.0001). Of 191 females, 139 (73%) received prostheses with nominal sizes 19 or 21 mm as compared to 26 (15%) out of 170 males (p<0.0001).

**Figure 1:**
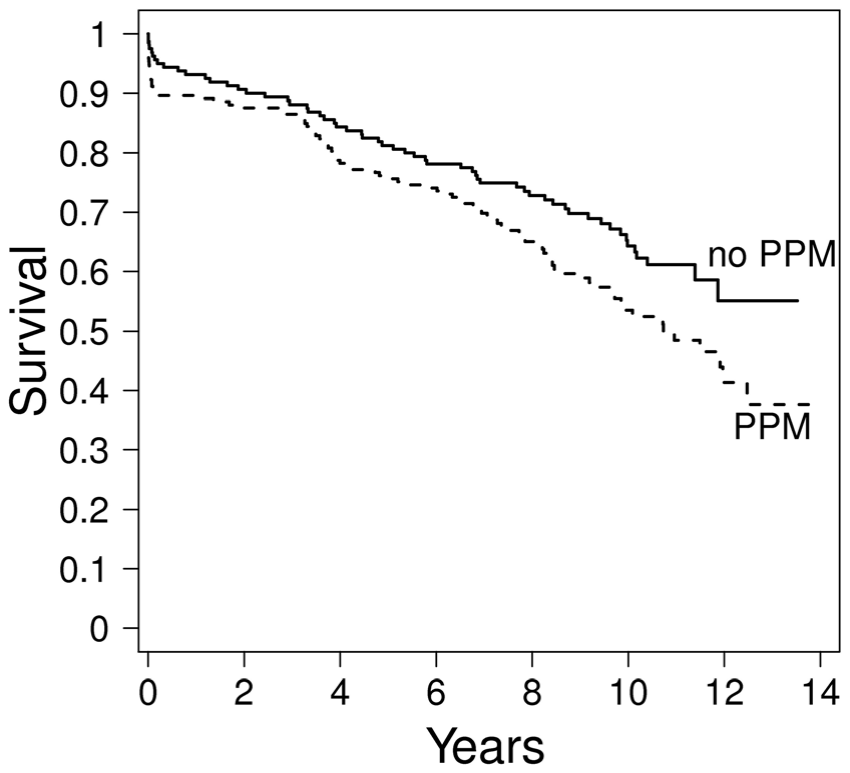
Kaplan Meier plot demonstrating overall survival according to presence or absence of patient-prosthesis mismatch (PPM).

Postoperatively, 172 patients (48%) were diagnosed with PPM, which was predictive of long-term survival (Figure 1). Table 2 compares baseline characteristics of patients with and without PPM. On an average, patients with PPM were older, had higher BSAs and were predominantly female. As expected, they had smaller aortic annulus and aortic root diameters, smaller LVs and a higher degree of left ventricular hypertrophy. A significantly higher proportion of PPM-patients could be identified among bioprosthetic valve carriers (145 out of 261, 56%) than among patients with mechanical valve prostheses (27 out of 100, 27%, p<0.0001).

**Table 2:** Clinical and echocardiographic characteristics of study patients.

	All patients(n = 361)	No PPM(n = 189)	PPM(n = 172)	p-value
**Clinical variables**				
Age [years]	69.5±10.2	66.9±11.0	72.2±8.4	<0.0001
Gender Female [n (%)]	191 (100)	68 (41.9)	123 (58.1)	<0.0001
Male [n (%)]	170 (100)	109 (64.1)	73 (35.9)	<0.0001
Height [cm]	167.12±8.85	167.34±9.09	166.88 ±8.60	= 0.6254
Weight [kg]	76.54±14.58	74.10 ±15.92	79.22±13.29	= 0.0007
BSA [m^2^]	1.88±0.21	1.85±0.22	1.91±0.19	= 0.0043
**Aortic stenosis variables**				
AVA [cm^2^]	0.61±0.17	0.62 ±0.17	0.61±0.18	= 0.7845
Mean AV gradient [mmHg]	65.57±19.54	64.62±20.41	66.61±18.56	= 0.3371
Aortic jet velocity [m/s]	4.99±0.77	5.04±0.75	4.94±0.78	= 0.2300
**LV and aortic geometry**				
Aortic annulus diameter [mm]	20.31±2.28	20.74±2.42	19.81±2.01	= 0.0002
LV wall thickness [mm]	16.19±2.76	16.05±2.89	16.37±2.59	= 0.3498
LV diameter [mm]	48.14±7.11	48.88±8.16	47.31±5.62	= 0.0461
LVEDD [mm]	49.40±7.52	49.72±10.37	48.38±5.91	= 0.1070
LVESD [mm]	30.29±7.35	30.76±9.22	29.37±5.89	= 0.1440
Aortic root diameter [mm]	32.97±4.69	33.95±5.08	31.81±3.90	<0.0001
**Aortic prostheses variables**				
Peak velocity [m/sec]	2.79±0.58	2.56±0.49	2.97±0.60	<0.0001
Mean gradient [mmHg]	18.34±8.08	15.88±6.57	20.65±8.69	<0.0001
EOA [cm^2^]	1.60±0.38	1.83±0.36	1.34±0.17	<0.0001
EOA/BSA [cm^2^/m^2^]	0.85±0.19	0.99±0.15	0.70±0.06	<0.0001
Bioprostheses [n (%)]	261 (100)	116 (44.4)	145 (55.6)	<0.0001
EOA bioprostheses [cm^2^]	1.50±0.29	1.72±0.29	1.33±0.14	<0.0001
EOA/BSA bioprostheses [cm^2^/m^2^]	0.82±0.16	0.96±0.13	0.70±0.06	<0.0001
Mechanical valves [n (%)]	100 (100)	73 (73)	27 (27)	<0.0001
EOA mechanical valves [cm^2^]	1.84±0.46	2.02±0.38	1.36±0.26	<0.0001
EOA/BSA mech. valves [cm^2^/m^2^]	0.95±0.23	1.05±0.18	0.69±0.08	<0.0001

Values are expressed as mean±SD or number (%). PPM, patient-prosthesis mismatch; BSA, body surface area; AVA, aortic valve area; LV diameter, end-diastolic diameter of the left ventricle from apical four-chamber view; LVEDD, left ventricular end diastolic diameter from M-Mode, parasternal short axis view; LVESD, left ventricular end systolic diameter from M-Mode, parasternal short axis view; EOA, effective orifice area.

### Determinants of prosthesis size

Clearly, EOA increases with increasing valve size [12]–[19]. Therefore, our first aim was to identify local anatomical structures that may limit the size of valve implants. In the univariable analysis (Table 3), a series of anatomical structures, including the aortic annulus diameter, aortic root diameter measured at the sinotubular junction, LV diameter as well as the mean pressure gradient and aortic valve area were found to be associated with prosthesis size. Other parameters related to prosthesis size were age, BSA and gender. In the multivariable model (Table 3), only aortic annulus diameter, aortic root diameter, LV dimension and gender were found to be determinants of prosthesis size.

**Table 3:** Univariable and multivariable regression models for nominal prosthesis size.

Simple Regression Models			
Variable	Parameter Estimate	95% Confidence Intervals	p-value
Age [years]	–0.06235	–0.08085; –0.04421	<0.0001
Gender	2.11709	1.78716;2.44703	<0.0001
BSA [m^2^]	3.62547	2.76281;4.48812	<0.0001
AVA [cm^2^]	3.11399	1.94377;4.28421	<0.0001
Mean AV gradient [mmHg]	–0.01199	–0.02221; –0.00177	= 0.0216
Aortic annulus diameter [mm]	0.43740	0.35752;0.51728	<0.0001
Wall thickness [mm]	0.11301	0.02943;0.19659	= 0.0082
LV diameter [mm]	0.11243	0.08584;0.13903	<0.0001
Aortic root diameter [mm]	0.23896	0.20026;0.27765	<0.0001
Prosthesis type (mech/bio)	0.82843	0.39472;1.26214	= 0.0002
Multivariable Model			
Intercept	22.02303	21.76644;22.27962	<0.0001
Gender	0.90438	0.49370;1.31506	<0.0001
Aortic annulus diameter [mm]	0.16030	0.06671;0.25389	= 0.0009
LV diameter [mm]	0.04418	0.01769;0.07068	= 0.0012
Aortic root diameter [mm]	0.13198	0.08782;0.17615	<0.0001

AVA, aortic valve area; LV diameter, end-diastolic diameter of the left ventricle from apical four-chamber view.

### Determinants of patient-prosthesis mismatch

The following parameters were found to be associated with smaller EOA/BSA values in the univariable analysis (Table 4): advanced age, female gender, smaller aortic annulus and aortic root diameters as well as smaller LV diameters, and finally, the presence of a bioprosthetic valve. In the multivariable model (Table 4), however, only a smaller aortic root diameter and the presence of a bioprosthetic valve remained associated with smaller EOA/BSA values (R-square 0.167). Results were confirmed in a sensitivity analysis, where presence or absence of PPM was defined as a binary outcome variable (aortic root diameter: OR 0.928 [95% CI, 0.876–0.982]; p = 0.0097 indicating a higher risk of PPM for smaller aortic root diameter; bioprosthetic valve: OR 3.534 [95% CI, 1.941–6.452]; p<0.0001 indicating a higher risk of PPM with presence of bioprosthetic valve).

**Table 4:** Univariable and multivariable regression models for factors determining patient-prosthesis mismatch.

Simple Regression Models			
Variable	Parameter Estimate	95% Confidence Intervals	p-value
Age [years]	–0.00608	–0.00789; –0.00426	<0.0001
Gender	0.08262	0.04431;0.12092	<0.0001
AVA [cm^2^]	0.03721	–0.08014;0.15457	= 0.5332
Mean AV gradient [mmHg]	–0.00050	–0.00151;0.00051	= 0.3298
Aortic annulus diameter [mm]	0.02240	0.01349;0.03132	<0.0001
Wall thickness [mm]	–0.00035	–0.00920;0.00850	= 0.9377
LV diameter [mm]	0.00481	0.00191;0.00771	= 0.0012
Aortic root diameter [mm]	0.01243	0.00777;0.01710	<0.0001
Prosthesis type (mech/bio)	0.13470	0.09321;0.17619	<0.0001
Multivariable Model			
Intercept	0.82768	0.80244;0.85292	<0.0001
Aortic root diameter [mm]	0.00878	0.00410;0.01346	= 0.0003
Prosthesis type (mech/bio)	0.12649	0.07839;0.17459	<0.0001

AVA, aortic valve area; LV diameter, end-diastolic diameter of the left ventricle from apical four-chamber view.

There was a significant correlation between aortic root diameter and all other continuous influence variables, explaining why the latter lost their predictive value in the multivariable model (correlation with age: r = –0.37, p<0.0001; with aortic annulus: r = 0.50, p<0.0001, with LV diameter: r = 0.28, p<0.001). Also gender showed a strong correlation with type of prosthesis (Chi square test: p<0.0001) and aortic root diameter (t-test: p<0.0001) and did not enter the final model.

**Figure 2:**
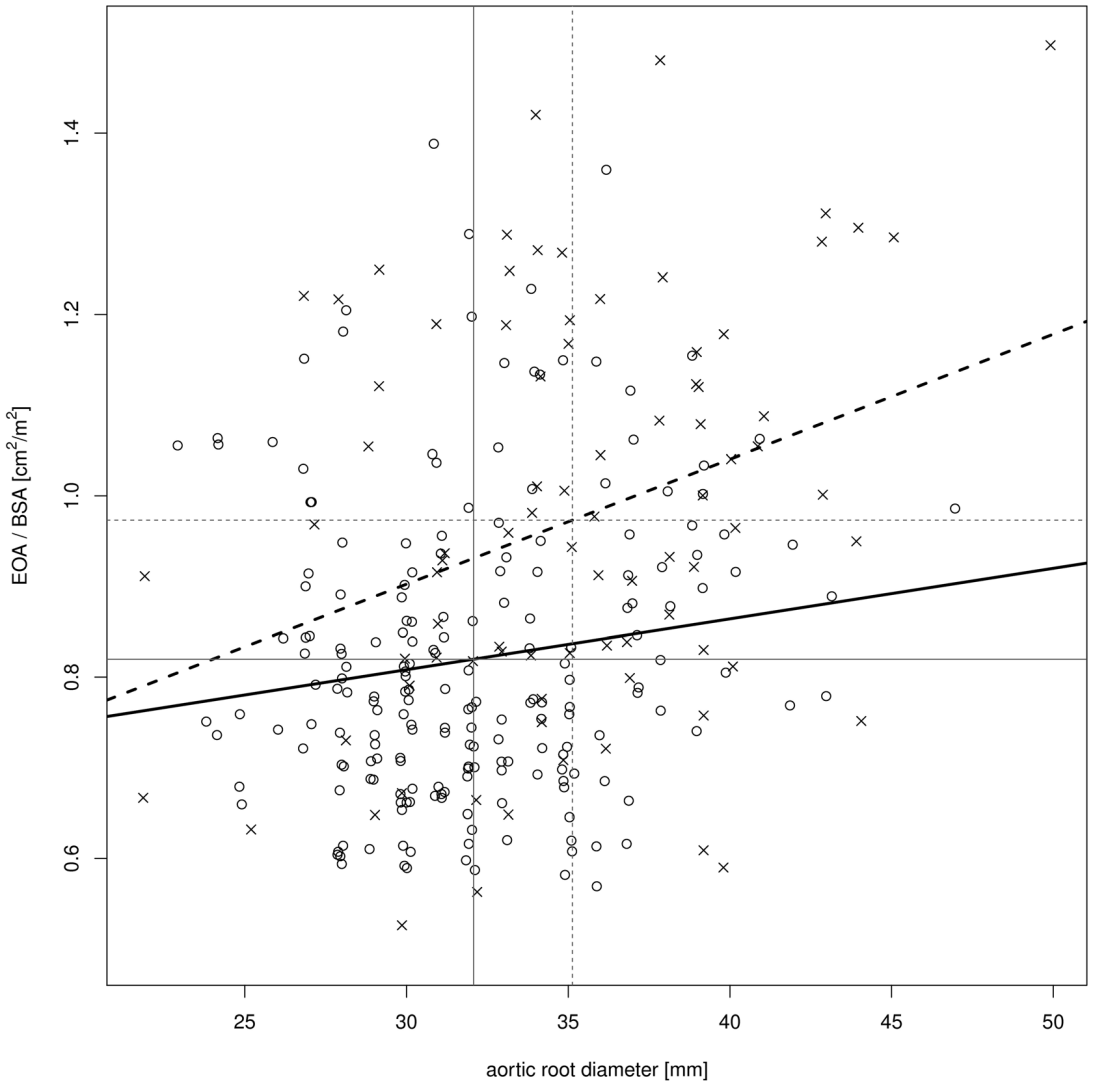
Scatterplot demonstrating the association between aortic root diameter and EOA/BSA separately for mechanical and biological valve grafts. By the use of mechanical prostheses, PPM is avoided to a larger extent when compared with bioprostheses. Crosses give values for mechanical prostheses and circles for biological prostheses. Bold lines show the regression lines separately for mechanical (dashed line) and biological prostheses (solid line). Horizontal lines show the mean values of EOA/BSA, separately for mechanical (dashed line) and biological prostheses (solid line). Vertical lines show the mean values of the aortic root diameter, separately for mechanical (dashed line) and biological prostheses (solid line).

Figure 2 illustrates the relation between PPM, the aortic diameter at the sinotubular junction and the type of prosthesis. Inclusion of a multiplicative interaction term between type of prosthesis and aortic root diameter into the multivariable model confirmed a differential impact of mechanical and biological valve prostheses (p = 0.092) on PPM occurrence. For an increasing aortic root diameter, PPM can be avoided to a larger extent by the use of mechanical valves as compared to bioprosthetic valves.

## Discussion

The issue of choosing an appropriate aortic prosthesis for an individual patient has received growing attention over the recent years. Particularly, the phenomenon of “patient-prosthesis mismatch” and its impact on morbidity and mortality after AVR has intensively been studied [5]. Although the importance of an adequately sized aortic prosthesis for operative and long-term outcome has been pointed out, factors favouring undersizing have not been studied systematically.

To identify determinants of prosthesis size and PPM, we studied a cohort of 361 consecutive patients undergoing AVR for isolated severe AS. In line with current literature that estimates PPM prevalence between 20% and 70% [1], approximately one half of our patients were affected (Table 2). Our main findings were that beyond local geometric dimensions, including diameters of the aortic annulus, aortic root and LV, female gender [20] was associated with smaller valve implants (Table 3). However, the likelihood of PPM occurrence was only determined by prosthesis type and the diameter of the aortic root at the sinotubular junction (Table 4).

Our data support previous recommendations on the use of mechanical valve prostheses to avoid PPM. For a given external valve diameter, conventional stented bioprosthetic valves will have smaller internal diameters and smaller EOAs, when compared with mechanical prostheses or stentless valve models [1]. Based on risk and benefit of mechanical versus bioprosthetic valves, current practice guidelines recommend the use of bioprosthetic valves in patients over the age of 65 years and in those who do not wish to take oral anticoagulation. Prosthesis type choice in our series was in adherence with current guidelines [8]. As a reflection of current surgical practice, only a minority of bioprosthetic valve carriers in our cohort had received a stentless valve (23 out of 261, 9%). In fact, this number was too small to yield statistically or clinically relevant conclusions with respect to PPM prevention in this specific subgroup.

The second predictor of PPM in our model was the aortic root diameter measured at the site of sinotubular junction. Surprisingly, the dimension of the aortic annulus, which is the major determinant for sizing of aortic prostheses, failed to predict PPM in the multivariable model. As an explanation we found a strong correlation between the diameters at the aortic annulus and those measured at the sinotubular junction (r = 0.50, p<0.0001). Moreover, when the aortic root diameter was omitted from the regression analysis, presence of a bioprosthetic valve (OR, 3.344 [95% CI, 1.887–5.917]; p<0.0001) and aortic annulus diameter (OR, 0.877 [95% CI, 0.786–0.979]; p = 0.0194) remained predictors of PPM. Although not independent from other aortic segments, the sinotubular narrowing may have an additional impact on the choice of graft size and PPM. Adhering with the established technique of aortic valve replacement, the ascending aorta is usually incised 1 to 2 cm above the sinotubular junction. After excision of the native aortic valve, the aortic valve graft is advanced trough the incised aorta, and after passing through the sinotubular junction it is sutured to the supraannular plane. Anatomically, the dimension of the sinotubular junction may become a bottleneck that hampers the passage of the aortic valve graft and limits prosthesis size [7], [8]. Nevertheless, aortic root enlargement propagated to prevent moderate PPM is currently not recommended as standard procedure, given prolonged aortic clamp times and increased operative mortality [21]–[23]. However, transcatheter aortic valve implantation (TAVI) may be an alternative in such cases, because prosthesis size is not limited by the aortic root diameter in this type of procedure.

A higher frequency of female patients with PPM has been reported in many studies from Europe, the US, Canada and Japan [5]. In accordance, we found 58% of female but only 36% of male patients (p<0.001) developing PPM. While in the univariable model female gender was clearly a predictor of PPM, it lost its influence in the multivariable model. This is explained by the fact that, on an average, women had smaller aortic root diameters than men (30.5±4.7 mm versus 35.3±4.2 mm, p<0.0001) and were more likely to receive bioprosthetic valves (156 (82%) versus 105 (62%), p<0.0001). Thus, anatomical and age-related factors rather than female gender itself seem to be associated with PPM.

### Limitations

Presented data have been collected in a single center setting. Therefore, a center-specific bias cannot be excluded, and all results and conclusions should be interpreted with caution. However, the major advantages of limiting data collection to a single center are 1. inclusion of a homogenous patient population, 2. adherence to a constant clinical routine, 3. constant quality of echocardiographic work-up and 4. constant surgical quality over a time-period of six years. Furthermore, the present study was dedicated to a specific subgroup of patients, namely those who underwent AVR for isolated severe AS. We are therefore unable to draw any conclusions on PPM occurrence in patients undergoing AVR for other reasons.

Our results may be influenced by a large proportion of patients who received bioprosthetic models with small EOA. It cannot be excluded that the use of bioprosthetic valves with larger EOAs may have yielded other results. Furthermore, the use of many different vales types with different hemodynamic properties limits the interpretation of the data.

Only 8 patients underwent aortic root enlargement. However, more complex surgical procedures to avoid PPM like aortic root enlargement put patients at additional risk by prolonging aortic clamp time and cardiopulmonary bypass time [21], [24]. It has also been shown that aortic annulus enlargement increases operative mortality [21], [25].

Despite its limitations, this is the first study that systematically analysed patient- as well as prosthesis-related parameters as potential determinants of PPM occurrence.

## Conclusions

From a clinical perspective, in particular female patients are at increased risk for PPM. This is explained anatomically, by the fact that they have smaller aortic roots, and demographically, by the fact that they are older when undergoing AVR and are therefore more likely to receive bioprosthetic valve grafts.

At present, the issue of PPM cannot be resolved by TAVI, where PPM rates of 18–35% have been reported [26], [27]. Furthermore, the natural history of calcified aortic valve leaflets that are compressed and displaced during the TAVI procedure is still unknown. Another unresolved issue is the serious problem of moderate to severe periprosthetic leakage after TAVI. However, it depends on future development whether TAVI will possibly overcome these technical and anatomical limitations and finally reduce PPM rates [28]. One should also bear in mind that the diagnosis of PPM may result from high echo gradients that cannot always be reproduced by invasive hemodynamics. Currently, novel sutureless bioprostheses with optimized hemodynamic performance seem to be a promising alternative to conventional bioprosthetic valves [25].

## Supporting Information

Checklist S1 STROBE Statement—Checklist of items that should be included in reports of *cohort studies.*
(DOC)
